# Deep-learning-based inverse design of three-dimensional architected cellular materials with the target porosity and stiffness using voxelized Voronoi lattices

**DOI:** 10.1080/14686996.2022.2157682

**Published:** 2023-01-04

**Authors:** Xiaoyang Zheng, Ta-Te Chen, Xiaoyu Jiang, Masanobu Naito, Ikumu Watanabe

**Affiliations:** a Graduate School of Pure and Applied Sciences, University of Tsukuba, Tsukuba, Japan; b Research Center for Structural Materials, National Institute for Materials Science, Tsukuba, Japan; c Department of Engineering Mechanics and Energy, University of Tsukuba, Tsukuba, Japan; d Research and Services Division of Materials Data and Integrated System (MaDIS), National Institute for Materials Science, Tsukuba, Japan

**Keywords:** Architected materials, inverse design, generative adversarial network, mechanical properties, finite element simulation, Voronoi lattices

## Abstract

Architected cellular materials are a class of artificial materials with cellular architecture-dependent properties. Typically, designing cellular architectures paves the way to generate architected cellular materials with specific properties. However, most previous studies have primarily focused on a forward design strategy, wherein a geometry is generated using computer-aided design modeling, and its properties are investigated experimentally or via simulations. In this study, we developed an inverse design framework for a disordered architected cellular material (Voronoi lattices) using deep learning. This inverse design framework is a three-dimensional conditional generative adversarial network (3D-CGAN) trained based on supervised learning using a dataset consisting of voxelized Voronoi lattices and their corresponding relative densities and Young’s moduli. A well-trained 3D-CGAN adopts variational sampling to generate multiple distinct Voronoi lattices with the target relative density and Young’s modulus. Consequently, the mechanical properties of the 3D-CGAN generated Voronoi lattices are validated through uniaxial compression tests and finite element simulations. The inverse design framework demonstrates potential for use in bone implants, where scaffold implants can be automatically generated with the target relative density and Young’s modulus.

## HIGHLIGHTS

- An inverse design framework for 3D Voxelized geometries using a 3D conditional generative adversarial network (3D-CGAN) is proposed.

- The trained 3D-CGAN is capable of generating 3D Voronoi lattices with the desired target relative density and Young’s modulus.

- The mechanical properties of 3D-CGAN generated Voronoi lattices were experimentally and numerically validated.

## Graphical Abstract

**Figure:**
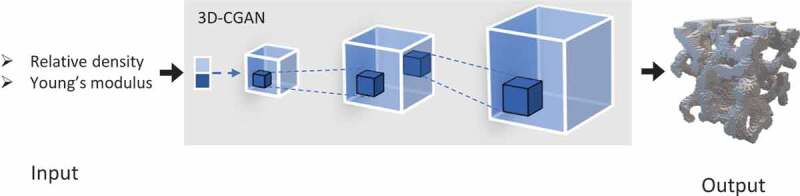

## 1. Introduction

Architected cellular materials are composed of cellular architectures designed within specific spatial arrangements [1–5]. Tailoring their microstructures offers a new architectural degree of freedom, which surpasses that of their constituent materials and results in the realization of unprecedented properties. Typical architected cellular materials include lattice materials with ultrahigh stiffness-to-weight ratios [6–8], negative Poisson’s ratio metamaterials [9–11], architected materials with shape reconfigurability [12,13], and metamaterials with programmable mechanical responses [14–17], facilitating their description as ‘metamaterials’. More importantly, recent advances in additive manufacturing have enabled the fabrication of complicated structures using multimaterials [18,19], shape memory polymers [20–22], and magnetic materials [23,24]. Thus, the rational design of architected cellular materials makes them promising candidates for soft robotics [18,23–25], actuators [16,17,26], soft electronics [27,28], tissue engineering [29–32], and electrochemical energy storage and conversion [33,34].

Over the last few decades, extensive efforts have been dedicated to the design of new architected cellular materials to achieve characteristics such as programable mechanical responses [14–17], novel deformation mechanisms [12,13,35,36], and theoretical stiffness and strength limits [6–8,37]. Notably, most of these studies have adopted the forward design, that is, a structure is designed based on computational modeling methods, and its effective properties are explored using time-consuming simulations and/or experiments. Using such forward design methods, models can be generated via mathematical modeling [10,31,32], Boolean and lofting operations [7–9,12,13], and topology optimization [38,39]. However, this requires experienced designers and extensive trial-and-error efforts to achieve the desired properties. Consequently, the forward design approach hinders practical applications to some extent. For instance, in tissue engineering, bone implants should be chosen to mimic damaged bones in terms of their biocompatibility, relative density (i.e. the volume fraction of solid part), and stiffness [29–32,40]. In such situations, the desired approach is the inverse design method, based on which implants can be designed and generated according to target properties and specific requirements.

In recent years, deep learning algorithms have been exploited to handle these inverse design challenges [11,41–50]. However, the direct generation of pixel-based representative volume elements – which take advantage of variational autoencoders and generative adversarial networks (GANs) — focuses primarily on two-dimensional geometries [11,44–47]. Although the inverse design of three-dimensional (3D) geometries has been successfully accomplished in some studies, the associated neural networks are always combined with additional modeling processes [41–43]. For example, in a recent study on the inverse design of truss metamaterials, the neural network was trained to output elementary lattices from existing datasets and their tessellations, which could be used to generate new truss metamaterials via geometric transformations [43]. In another study on the inverse design of spinodoid metamaterials, the neural network was trained to output design parameters that could be used to generate a topology via the linear Cahn – Hilliard model [41]. Notably, these studies adopted an indirect approach to generate such complex geometries: the trained neural networks generated modeling parameters that could be used to create geometries based on additional modeling procedures. Here, by contrast, we employ the GAN to directly generate 3D voxel-based representative volume elements (RVEs) (i.e. voxelized Voronoi lattices) without the need for an additional modeling process. Voronoi lattices are disordered architected materials; the irregularity not only makes their morphology similar to that of bones but also broadens their diversity in terms of the stiffness and strength for a given relative porosity.

In this study, we developed a deep-learning-driven inverse design framework for the direct generation of 3D voxelized architected cellular materials with user-defined relative densities (ρ) and Young’s moduli (E). The adopted inverse design framework is a 3D conditional GAN (3D-CGAN) based on volumetric convolutional neural networks. The 3D-CGAN was trained using a dataset consisting of 10,000 3D Voronoi lattices and their labels (ρ and E). The lattices were derived from the Voronoi tessellation, and their Young’s moduli were calculated using a numerical homogenization algorithm. The trained 3D-CGAN used the target relative density and Young’s modulus as the inputs, and it output the corresponding 3D voxelized Voronoi lattices. The mechanical properties of the generated Voronoi lattices were verified using uniaxial compression tests and finite element method (FEM) simulations.

## 2. Methods

### 2.1. Dataset preparation

**Figure 1.**
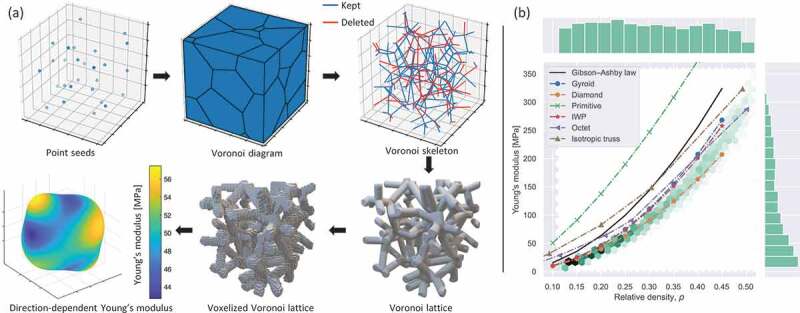
Dataset preparation. (a) Process of the dataset preparation: the geometry of a voxelized Voronoi lattice was generated using Voronoi tessellation and its Young’s modulus was calculated using the homogenization method. (b) Dataset consisting of 10,000 datapoints where each datapoint was composed of a geometry (a 3D voxel array with a shape of [64,64,64]), its relative density, and Young’s modulus. The data space was compared with several typical architected materials, including Gyroid, Schwarz Diamond, Schwarz Primitive, and Schoen IWP lattices, and octet and isotropic trusses [37,52].

**Figure 2.**
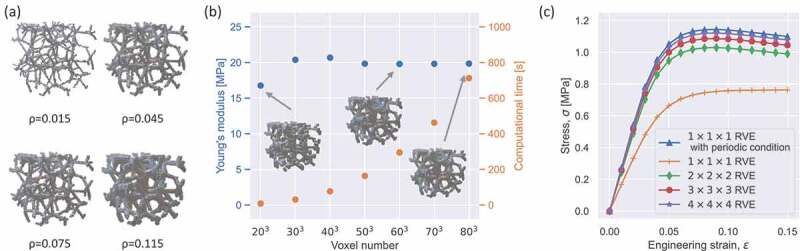
Assessing modeling, numerical homogenization algorithm, and FEM simulation. (a) Voxelizing Voronoi lattice with different relative densities. (b) Computational accuracy and cost of the numerical homogenization algorithm. The Voronoi lattice was voxelized into 3D voxel arrays with different shapes. (c) Effect of the RVE size on the computational accuracy of FEM simulations. The Voronoi lattice was modeled with different RVE sizes, where n×n×n RVE means an RVE consisting of n×n×n unit cell numbers.

Figure 1a shows the process of generating 3D Voronoi lattices for dataset preparation. First, a random seed of 27 3D coordinate points was created using Mitchell’s best-candidate algorithm [51]. Note that the algorithm generates coordinate points with a regular distribution. Thereafter, a Voronoi diagram was plotted using Laguerre – Voronoi tessellation with a 3D periodic boundary condition. The periodic condition was implemented. The seeds were generated in 3×3×3 unit cells and the center unit cell was defined as an RVE. The same approach was employed for 2D design in our previous study [17]. The Voronoi skeleton was derived from the polyhedral meshes of the Voronoi diagram. It should be noted that nodal connectivity, which refers to the total number of ligaments connected to a node, has a considerable impact on the stiffness of architected materials [52]. For example, ordered architected materials with nodal connectivities of 3, 4, 6, and 8 are significantly different in terms of their Young’s modulus, yield strength, and Poisson’s ratio [52]. Changing the nodal connectivity may increase the diversity of Young’s modulus for Voronoi lattices with the same relative density. Therefore, to extend the border of the available data space, 0–30% of the edges in the polyhedral meshes were deleted randomly to change the nodal connectivity. Consequently, 30% was chosen to maintain the isotropy of the Voronoi lattices while extending the boundary of the available data space. Note that if this value is too large, the Voronoi lattices will become anisotropic. Also, the appearance frequency of each node keeps over 1 to prevent single element connectivity when randomly deleting edges. Additionally, a periodic boundary condition was applied in this process to ensure the periodicity of the generated Voronoi lattices. Finally, a triply periodic Voronoi lattice was generated after a specific thickness was assigned to the edges of the polyhedral meshes. For deep learning, each Voronoi lattice was voxelized into a 3D voxel array ($A_{v}$) with a shape of [64,64,64]. To investigate the lower volume fraction applicability limit, the relative density of a Voronoi skeleton was reduced. The Voronoi skeleton was voxelized with decreasing relative densities, and the result revealed that the voxelized Voronoi lattice became discrete if the relative density was less than 0.045 (Figure 2a). Considering a margin, the minimum relative density of the Voronoi lattices was set to 0.1. The modeling process was implemented using a Python code, and the Laguerre – Voronoi tessellation was based on the Python package MicroStructPy [53].

The Young’s moduli of the generated 3D Voronoi lattices were calculated using a numerical homogenization method, as detailed in past studies [54–56]. That is, the input argument was a 3D voxel array consisting of 0 and 1, where 1 indicates a solid, and 0 indicates a void. The Young’s modulus and Poisson’s ratio of the constitutive materials were set as 1.6 GPa and 0.23, respectively, corresponding to the material parameters of a 3D-printed resin. Periodic boundary conditions were then applied during the homogenization process. The homogenized constitutive matrix $C^{H}$ could be solved by obtaining the element displacements and global displacement field, as follows:

(1) $$\begin{array}{c} C_{ij}^{H}=\frac{1}{\left|V\right|}\sum_{\left(e\right)}\int_{V^{\left(e\right)}}{\left(\chi_{\left(e\right)}^{0(i)}-\chi_{\left(e\right)}^{\left(i\right)}\right)}^{T}k_{e}\left(\chi_{\left(e\right)}^{0(\;j)}-\chi_{\left(e\right)}^{\left(\;j\right)}\right)dV^{\left(e\right)} \end{array}$$

where $\left|V\right|$ denotes the total volume of the cube domain, $\chi_{\left(e\right)}^{0(i)}$ denotes the element displacement, $\chi_{\left(e\right)}^{\left(i\right)}$ denotes the displacement field obtained from the global stiffness equation, and $k_{e}$ denotes the element stiffness matrix. Following iterations for all six load cases (three compressions along the x, y, and z axes, and three shearing loads), the effective 6×6 elasticity matrix $C^{H}$ was obtained. The directional dependence of the Young’s modulus of a typical Voronoi lattice is shown in Figure 1, where the shape of the surface contour is close to a sphere, thus indicating the approximate isotropic stiffness of the Voronoi lattice. Consequently, the effective Young’s modulus can be obtained using isotropic approximation, where the complete 6×6 elasticity matrix is matched with the matrix for the isotropic symmetry class. It should be noted that the effective Poisson’s ratio can also be calculated using the elasticity matrix. The accuracy of the Poisson’s ratio computed by the numerical homogenization method in our previous work [11]. Because the Voronoi lattices were approximated as voxel arrays rather than beam-based architected materials, we conducted a convergence study according to the number of voxels and computational cost. A Voronoi lattice (ρ=0.115) was voxelized into 3D voxel arrays with different shape from [20,20,20] to [80,80,80]. The Young’s moduli of such 3D voxel arrays were calculated using the numerical homogenization method. The calculated Young’s moduli and computational cost are compared in Figure 2b. From the figure, it can be observed that the Young’s modulus converges with the increase in the number of voxels, and the value remains constant when the number of voxels is greater than [50,50,50]. Further, the computational cost (code execution time on a MacBook with M1 chip) increases exponentially with the number of voxels. Therefore, with regard to the computational accuracy and cost, Voronoi lattices were voxelized into 3D voxel arrays with a shape of [64,64,64], which corresponds to $64^{3}$ eight-node hexahedral elements in the numerical homogenization method.

The relative density (ρ) refers to the proportion of the solid part in a Voronoi lattice and can be calculated as follows:

(2) $$\begin{array}{c} \rho =\frac{\sum A_{v}}{64^{3}} \end{array}$$

Figure 1b shows the data space of material properties for 10,000 generated Voronoi lattices in terms of the ρ–E relationship, where the darker region indicates a higher concentration of datapoints. The material property space was compared with that of typical architected materials [37,52]. Notably, the relationship between ρ−E does not follow the classical scaling laws of the Gibson – Ashby model ($E^{*}/E_{0}=a\rho^{b}$, where $E^{*}$ denotes the effective Young’s modulus, $E_{0}$ denotes the Young’s modulus of the constituent material, and a and b are constants) [57]. By contrast, the data space of ρ and E covers a wider range, exhibiting a ribbon pattern. This implies that neural networks can be trained to generate Voronoi lattices with corresponding properties inside the ribbon-shaped data space. Overall, the training dataset comprises 10,000 datapoints, where each datapoint consists of a Voronoi lattice and its corresponding ρ and E.

In the numerical homogenization method, numerous unit cells are considered based on a mathematical operation [58]. However, the number of unit cells is very limited in an experimental validation. Therefore, the effect of the number of RVE units on stress – strain curve at finite strain was investigated for a comparison with the corresponding experiment as shown in Figure 2c. The results are addressed in Section 2.4.

### 2.2. 3D conditional generative adversarial network

The neural network architecture yields two main outputs that are necessary for the inverse design of mechanical metamaterials: the modeling parameters, which can be used to generate geometries with additional modeling processes [41–43], and the geometries in the form of pixels or voxels [11,46,47,59,60]. The straightforward generation of geometries can speed up the inverse design process and directly visualize geometries. The variational autoencoder (VAE) and GANs are the most commonly used neural network architectures for straightforward generation [47,59,60]. In the VAE, an encoder learns to represent input data (e.g. geometry or modeling parameters) efficiently, and a decoder tries to reconstruct the data using the internal representations and the learned weights, making it an ideal data compression engine. By contrast, the GAN is trained in an adversarial feedback loop to generate realistic geometries, adopting variational sampling to generate distinct geometries [11,46]. Consequently, the GAN may be superior to the VAE in terms of the generation performance. We compare relevant studies using different network architectures for the inverse design of mechanical metamaterials in Table 1.

**Table 1.** Comparison of neural networks for the inverse design of mechanical metamaterials.

Input	Output	Geometry type	Neural network type	Number of training datapoints	R-square	Relative error	MSE	Reference
Stiffness tensor	Modeling parameters	3D spinodoid metamaterials	Multi-layer perceptron	19,170	0.999			[41]
Stiffness tensor	Modeling parameters	3D truss metamaterials	Multi-layer perceptron	3,000,000	0.986			[43]
Elastic modulus and relative density	Modeling parameters	2D honeycomb, square, and re-entrant star-shaped lattices	Multi-layer perceptron	53,000		0.05%		[48]
Stress–strain curve	16 binary representation of geometric infills	2D checkerboard-shape non-uniform cellular materials	Multi-layer perceptron	16,576			0.00031	[45]
Filter radius, volume fraction, and a design objective (maximum bulk modulus, maximum shear modulus, or minimum Poisson’s ratio).	128 × 128 pixels	2D metamaterials	Variational autoencoders	25,000			0.009	[47]
Young’s modulus and Poisson’s ratio	256 × 256 pixels	2D auxetic metamaterials	Generative adversarial network	100,000			0.014	[11]
Relative density and Young’s modulus	64 × 64 × 64 voxels	3D disordered voxelized lattices	3D Generative adversarial network	10,000			0.01	This work

In this study, the inverse design of 3D architected cellular materials was implemented using a novel framework: 3D-CGAN. The 3D-CGAN was trained to generate 3D voxels of Voronoi lattices from a probabilistic space with a given label (i.e. ρ and E) by leveraging recent advances in controllable GANs and volumetric convolutional networks [61–64]. The 3D-CGAN had a structure similar to that of the neural network used in our previous study, wherein 2D auxetic metamaterials were generated using a CGAN [11]. In this study, we improved the neural network (3D-CGAN) and enhanced its ability to generate 3D geometries according to the target properties. The 3D-CGAN comprised three modules: a generator, a discriminator, and a calculator, which were trained by an adversarial process. Notably, the generator learns to generate 3D voxelized Voronoi lattices that mimic the geometries of the real Voronoi lattices from the dataset. The discriminator learns to distinguish real Voronoi lattices (from dataset) from fakes (generated by the generator), and it thus helps generate realistic Voronoi lattices. The calculator learns to predict ρ and E of given Voronoi lattices, and it thus helps the generator spawn Voronoi lattices with the desired target properties.

**Figure 3.**
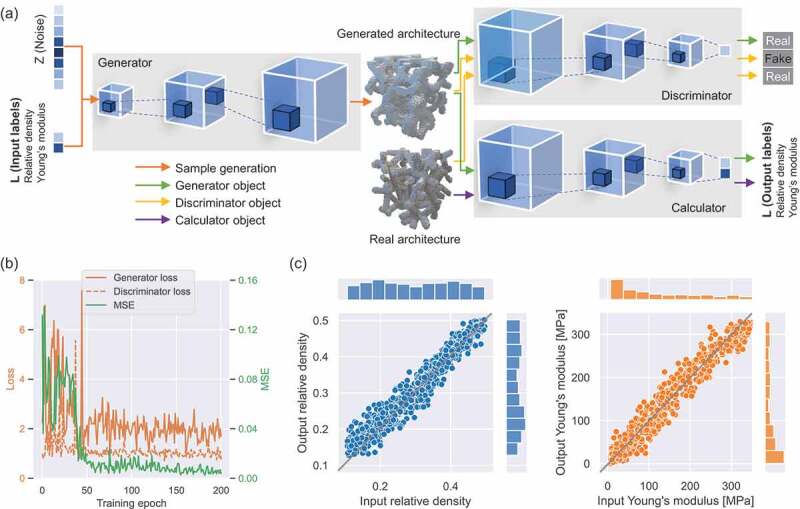
3D-CGAN framework and performance. (a) 3D-CGAN comprising three modules: generator, discriminator, and calculator. (b) Generator loss, discriminator loss, and mean square error against training epoch. (c) User-input against 3D-CGAN-output values for the relative density and Young’s modulus.

Figure 3a shows the framework of the 3D-CGAN. The training process is based on supervised learning. While training, the generator progressively becomes better at creating Voronoi lattices that look real and exhibit the desired target properties, whereas the discriminator becomes better at distinguishing between real and fake Voronoi lattices. The process attains equilibrium when the generator can perfectly deceive the discriminator. The 3D-CGAN has the ability to generate a batch of 3D voxelized Voronoi lattices for a given label (ρ and E) after being trained using 10,000 datapoints. The detailed information of the 3D-CGAN is discussed in Appendix A. Overall, the three primary advantages of the 3D-CGAN are as follows: first, compared with traditional heuristic criteria (e.g. the genetic algorithm), the use of an adversarial criterion speeds up the inverse design process and enables the generator to capture the object structure implicitly; second, the generator establishes the modeling process from a 1D probabilistic space to the 3D space of objects, without the use of an additional modeling process; and third, the calculator serves as an independent module that helps the discriminator avoid overfitting.

### 2.3. Uniaxial compression tests

**Figure 4.**
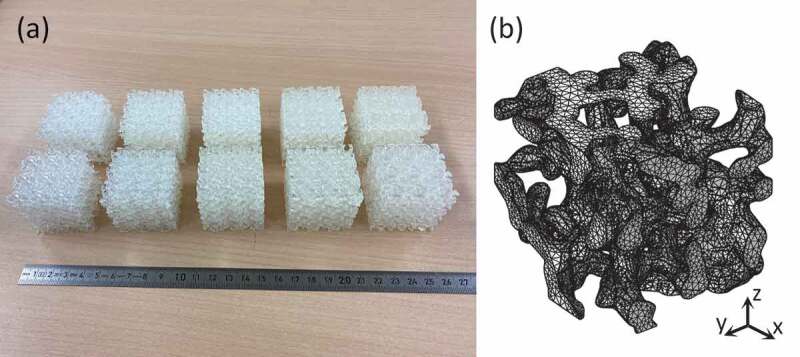
Visualization of Voronoi lattices. (a) Representative 3D-printed samples before (top) and after (bottom) surface smoothing. (b) Representative mesh used in the FEM simulations.

The mechanical properties of the 3D-CGAN generated Voronoi lattices were first investigated using uniaxial compression tests. Notably, to obtain the stiffness of these generated Voronoi lattices, 3D printing technology is ideal, which allows the fabrication of such complex models. To print models without supporting components, while providing smooth surface finishing, we used a stereolithography 3D printer (Form 3, Formlabs, USA) with a photopolymer resin (clear resin, Formlabs, USA). The photopolymer resin is a typical plastic material with a Young’s modulus, Poisson’s ratio, and yield strength of 1.6 GPa, 0.23, and 38 MPa, respectively [65]. The 3D-printed models had dimensions of 40 mm × 40 mm × 40 mm and comprised an RVE of 3×3×3 unit cells, which were selected to represent such types of periodic porous materials in terms of mechanical testing [52]. These models were exported as standard tessellation language (STL) format files and then sliced through PreForm before being sent for 3D printing. The printing parameters were set as follows: a layer thickness of 0.05 mm and an operating temperature of $33^{\circ }$C with no support structures. To remove the residual resin from the surface, these samples were washed with isopropanol after 3D printing. Thereafter, a post-curing process was implemented on these samples at $60^{\circ }$C for 30 min using Form Cure (Formlabs, USA). Figure 4a shows the representative 3D-printed samples before and after surface smoothing.

After 3D-printing fabrication, the mechanical properties of these samples were examined through uniaxial compression tests using a motorized test stand (AGXplus-10kN, Shimadzu, Japan). The static compression tests were performed at a vertically constant speed of 10 mm/min, following the ASTM standard D695–15. The compression strain was set to 0.15, which is adequate to achieve elastic deformation of these samples. The deformation processes were recorded using a high-speed camera placed in front of the samples. The effective Young’s moduli were calculated by linearly fitting the initial linear region of the recorded stress – strain curves.

### 2.4. Finite element method simulations

The deformation behavior was visually examined using FEM simulations. The deformation problem under a finite strain was analyzed using a nonlinear FEM simulation, where periodic boundary conditions were applied on a periodic microstructure. The detailed process of implementing the displacement and stress equations has been discussed in our previous reports [59,66]. FEM simulations were performed using a commercial FEM simulation platform (COMSOL Multiphysics Ver. 5.6, COMSOL, Sweden). The constitutive model was a plastic material model with a Young’s modulus, Poisson’s ratio, and yield strength of 1.6 GPa, 0.23, and 38 MPa, respectively, corresponding to the mechanical properties of the clear resin used in the experiments. Nonlinear uniaxial compression simulations were performed according to the periodic boundary conditions, accompanied by a parametric sweep of the z-axis displacement. The stop condition was set when the compression strain was 0.15. The models were built using approximately $3\times 10^{5}$ second-order tetrahedral solid elements, and a typical meshed model is shown in Figure 4b. The effective Young’s moduli were also extracted from the stress – strain curves.

In the experiments, uniaxial compression deformation was imposed to 3D-printed samples composed of 3×3×3 RVE units. To ensure the validity of the experiments, the effect of number of RVE units was investigated. Here, we computed the large deformation behavior of a Voronoi lattice (the same Voronoi lattice in Figure 2b with a voxel array of [64,64,64]) in the uniaxial compression test with different number of RVE units using FEM simulations. The stress – strain curves obtained from the FEM simulations are shown in Figure 2c. The figure shows that the stress – strain curves converge as the RVE size increases. It should be noted that the stress – strain curve of a 1×1×1 RVE with periodic condition fits with those of larger RVE sizes (e.g. 3×3×3), demonstrating that the experiments correspond to numerical simulations of an RVE with periodic conditions.

## 3. Results and discussion

### 3.1. Training results

The performance of the 3D-CGAN can be evaluated based on the similarity and stability of the training results. Here, similarity refers to the agreement between input labels (targets ρ and E) and output labels (ρ and E of the generated structures). We evaluated the similarity using a risk function, that is, the mean squared error (MSE) of the sum of ρ and E:

(3) $$\begin{array}{c} MSE=\frac{1}{n}\sum_{i=1}^{n}\left({\left(\rho_{i}-{\hat{\rho }}_{i}\right)}^{2}+{\left(E_{i}-{\hat{E}}_{i}\right)}^{2}\right) \end{array}$$

where n denotes the total number of labels sampled from the available ρ−E data space. To reduce the error, n was set to 1024 at each generation. ρ denotes the input relative density, $\hat{\rho }$ denotes the output relative density, E denotes the input Young’s modulus, and $\hat{E}$ denotes the output Young’s modulus. To stabilize the training process, the relative density and Young’s modulus were normalized to the range 0–1. A smaller MSE indicates better similarity between the input and output labels, as well as better performance of the 3D-GAN.

The stability refers to a stable training process. In the 3D-CGAN, the generator and discriminator models were trained simultaneously, with the goal of finding a Nash equilibrium between the two models. Consequently, the training process aims to find an equilibrium between two forces rather than a minimum. The stability can be quantitatively evaluated in terms of the discriminator and generator losses (see Appendix A for the definition).

Figure 3b displays the MSE and loss versus the training epoch using 10,000 datapoints. The MSE curve consists of two stages: the MSE decreases initially before gradually converging and finally attains a minimum at approximately 0.01 after epoch 50. This shows that the 3D-CGAN can be trained to converge after finite epochs. The low value of the MSE shows that the trained 3D-CGAN has learned to generate Voronoi lattices with a target ρ and E. The loss curves show the typical pattern of a reliable GAN training procedure, that is, both losses are slightly erratic early in the run before stabilizing after approximately 50 epochs. The losses converge to a stable equilibrium, proving the stability of the training process. The convergence of the MSE and losses demonstrates the robustness of the 3D-CGAN and the stability of the training process.

### 3.2. Inverse design of architected cellular materials

Given that the training results were robust and stable, we managed to exploit the trained 3D-CGAN for the controllable generation of Voronoi lattices. The inverse design adhered to the following procedure: the 3D-CGAN received a label (ρ and E) and then yielded several voxelized Voronoi lattices with the target ρ and E. To demonstrate the flexibility of the trained 3D-CGAN, Voronoi lattices were generated with target labels randomly selected from the ρ–E data space. Figure 3c compares the input labels (targets ρ and E) and output labels (ρ and E of the generated structures) of 1024 randomly generated Voronoi lattices. Each coordinate of the scatter corresponds to an input ρ or E and the output ρ or E. The difference between the input and output labels can be evaluated by linearly fitting these scatters (X=Y). As shown in Figure 3c, these scatters converge to the bisection line, forming a narrow region. The distributions of the user-input and 3D-CGAN-output values are also compared in Figure 3c. The mean values of the user-input and 3D-CGAN-output relative densities are 0.3068 and 0.3165, and the variances of the user-input and 3D-CGAN-output relative densities are 0.0132 and 0.0123, respectively. The mean values of the user-input and CGAN-output Young’s moduli are 125.1 and 123.5 MPa, and the variances of the user-input and CGAN-output Young’s moduli are 10332 and 10117 MPa, respectively. This indicates that the trained 3D-CGAN has learned to generate Voronoi lattices with the target ρ and E. In addition, these results also prove the successful implementation of controllable inverse design, making it unique from the forward design method, where Voronoi lattices are generated by Voronoi tessellation without assigned ρ and E.

**Figure 5.**
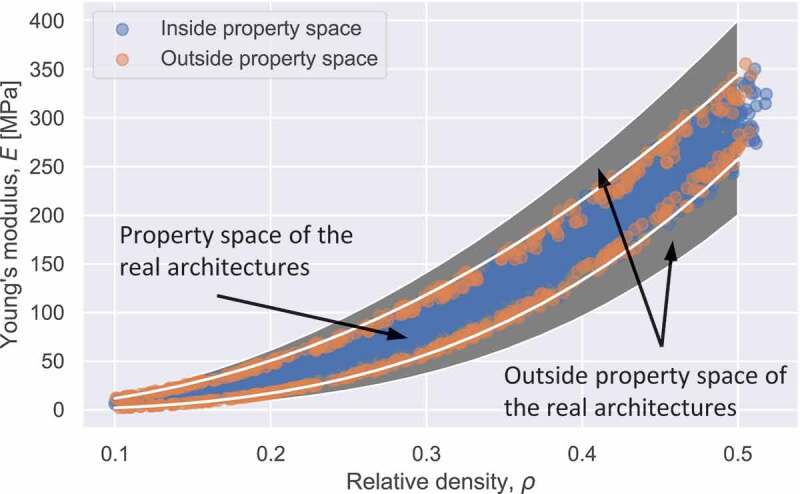
Properties of the 3D-CGAN-generated Voronoi lattices covering the relative density-Young’s modulus relationship map. Blue dots represent properties of 3D-CGAN-generated Voronoi lattices that were generated by inputting target properties inside the data space, and the orange dots represent those generated by inputting target properties outside the data space.

To entirely explore the capability and applicability of the 3D-CGAN, we compared the data spaces of the real Voronoi lattices and those of the 3D-CGAN-generated Voronoi lattices in the relative density – Young’s modulus relationship map in Figure 5. We first input the target properties inside the data space of the real Voronoi lattices to the 3D-CGAN, and we then plotted the properties of the 3D-CGAN-generated Voronoi lattices in Figure 5. The data space of the real Voronoi lattices refers to the ribbon region in the relative density – Young’s modulus relationship map in Figure 1b. It can be observed that the properties of the 3D-CGAN-generated Voronoi lattices can occupy the data space, further demonstrating that the 3D-CGAN possesses the ability to generate Voronoi lattices with properties akin to the dataset. To explore the capability of generating Voronoi lattices with properties outside the dataset, we tried to input target properties outside the data space of the real Voronoi lattices. The results revealed that the trained 3D-CGAN can barely generate Voronoi lattices with properties outside the data space but approaching the border of the property space, as shown in Figure 5. This can be attributed to the training target of the 3D-CGAN: the 3D-CGAN was trained to learn to generate Voronoi lattices that not only look real but also have the target properties. To achieve the target properties, the 3D-CGAN learned to deceive the calculator that was initially trained with the data space of the real Voronoi lattices. Consequently, it was difficult for the 3D-CGAN to generate Voronoi lattices in the whitespace beyond the data space.

**Figure 6.**
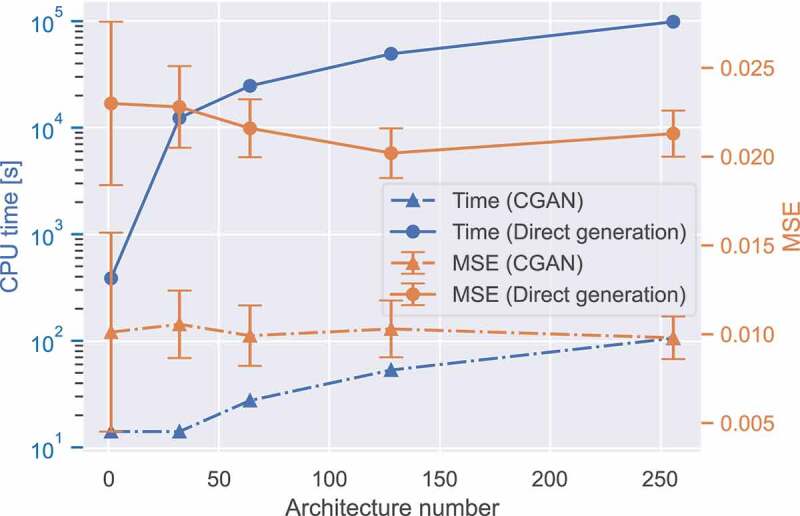
Computational cost and accuracy of the inverse design by 3D-CGAN and forward design. The computational cost was determined using the CPU time of code execution, and the accuracy was measured using the MSE based on the target property and the property of the generated geometries.

To demonstrate the benefits of the 3D-CGAN in terms of the computational cost and accuracy, we compared the generation processes of the inverse and forward designs. The inverse design was implemented using the trained 3D-CGAN to generate a given number of geometries with a target property (ρ=0.3 and E=90 MPa). The code execution time was measured according to the computational cost. For the forward design, it is clear that one can directly generate a large number of geometries using Voronoi tessellation and calculate their properties, and then select the desired geometries with target properties. The computational cost of the forward design was determined using the code execution time required for running the Voronoi tessellation and numerical homogenization. All codes were run on a MacBook with an M1 chip. Figure 6 compares the computational costs between the inverse and forward designs. The results show that the time require to execute the forward design on a central processing unit (CPU) is greater than that required for an inverse design based on the trained 3D-CGAN by a factor of 1000. For example, generating 128 Voronoi lattices 3D-CGAN requires approximately $5\times 10^{4}$ s using the forward design but only 53 s using 3D-CGAN, which is significantly faster than that required to generate optimized 3D geometries using Solid Isotropic Material with Penalization (SIMP) topology optimization [67,68]. It is clear that topology optimization can generate an optimized 2D geometry in a short time. However, a longer time is required to generate a batch of optimized 3D geometries using topology optimization than that required by the 3D-CGAN [69]. In addition, when multiple constraints (e.g. Young’s modulus, Poisson’s ratio, yield strength, and porosity) are required in topology optimization, the computational cost may be rapidly increased. However, the time can barely change for the 3D-CGAN because only the labels of the training dataset are to be replaced. We also calculated the MSE using the target property and the property of the generated geometries. The results show that the MSE of the 3D-CGAN-generated lattices is approximately 0.1, which is better than that for the forward design of randomly direct generation (around 0.21) (Figure 6).

**Figure 7.**
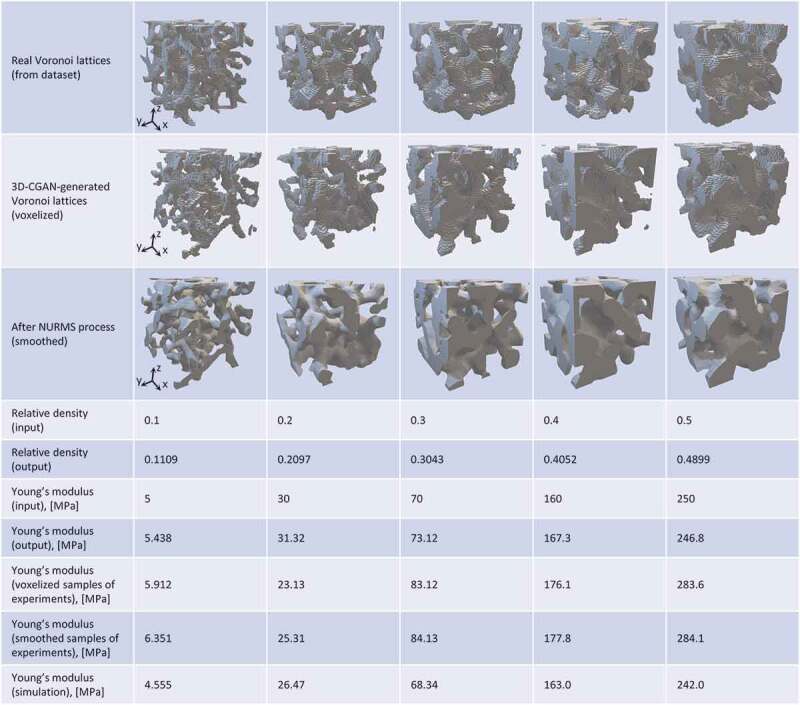
3D-CGAN generated Voronoi lattices with different relative densities and Young’s moduli before and after surface smoothing. The relative densities and Young’s moduli were validated through experiments and simulations.

To visualize the training results clearly, Figure 7 compares several real Voronoi lattices (generated using Voronoi tessellation) and 3D-CGAN-generated Voronoi lattices. Similar to real Voronoi lattices, these voxelized Voronoi lattices have a ligament-channel bicontinuous network. Additionally, the input and output relative densities and Young’s moduli show significant agreement, further demonstrating that the 3D-CGAN can generate Voronoi lattices with the target ρ and E. It should be noted that a few isolated voxel clusters could be found in some 3D-CGAN-generated Voronoi lattices, which can be attributed to the transposed convolution layers in the generator that are provided with random noise as an input. These isolated voxel clusters can be removed by filtering the isolated voxels after generation. The 3D-CGAN is trained in an adversarial feedback loop to generate realistic geometries, which indicates that the 3D-CGAN-generated Voronoi lattices appear realistic. As the 3D-CGAN-generated Voronoi lattices are generated from random noise, these Voronoi lattices are similar but distinct from the real geometries and themselves. In addition, for bone implant application, we smoothed the surface of the generated Voronoi lattices using the non-uniform rational mesh smooth (NURMS) method Figure 7).

### 3.3. Validation via experiments and simulations

**Figure 8.**
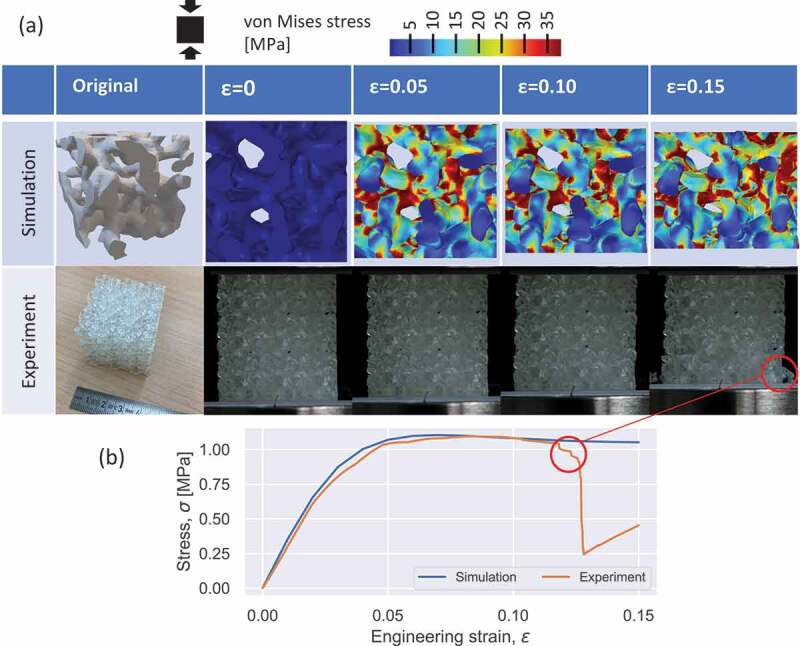
Deformation behavior of a 3D-CGAN generated Voronoi lattice. (a) FEM simulation and experimental results of the Voronoi lattice deforming under increasing compressive strain. (b) Stress–strain curves obtained from FEM simulations and uniaxial compression tests. The sudden drop in stress during compression corresponds to the local fractures appearing in the 3D-printed sample.

We further validated the mechanical properties and deformation behaviors of the 3D-CGAN generated Voronoi lattices using uniaxial compression tests. Figure 8a displays a generated Voronoi lattice after surface smoothing, showing a gradual deformation under a progressive compression strain. This suggests that some local fractures appeared under compressive loading (the red circles in Figure 8a). The ligament crack can be attributed to the local stress concentration owing to the geometrical irregularity and brittleness of the 3D-printed resin. These local fractures contribute to the sudden drop in the stress – strain curve compared to that obtained from the experimental compressive test (Figure 8b). This result suggests that the 3D-printed resin is not a suitable material for applications in scaffolds because of its brittleness, which is one of the reasons why many bone implants are fabricated with alloys [29,32,70,71].

However, the aim of using the 3D-printed resin in this study was to validate the stiffness. Consequently, the Young’s modulus of each sample was calculated from the linear elastic region of the stress – strain curves. The 3D-CGAN generated Voronoi lattices before and after the NURMS smoothing process were prepared and evaluated using compression tests. The calculated Young’s moduli of these samples are compared in Figure 7. Although a sample becomes slightly stiffer after surface smoothing (no more than 10%), the Young’s moduli calculated from the linear elastic regions showed a significant agreement with the target values, demonstrating the accuracy of the trained 3D-CGAN (Figure 7b). The results prove that the NURMS method can be combined with a CNN to generate smoothed geometries with target stiffnesses.

The mechanical properties were validated using FEM simulations. Compared with a 3D-printed sample consisting of 3×3×3 unit cells, the geometry in the FEM simulation had only one unit cell owing to the application of periodic conditions. Figure 8a shows the progressively deformed configurations of the smoothed Voronoi lattice. This demonstrates that the stress is concentrated in the middle regions of the ligaments, as well as the contact region of the surfaces. Moreover, based on a comparison between the experimental and simulation results, the stress – strain curves in the linear elastic region showed a significant agreement, demonstrating that the use of FEM simulations was a robust approach to predict the stiffness of such architected materials. Additionally, it is striking to note that the Young’s moduli obtained from the experimental and simulation results were close to the target values, further proving that the trained 3D-CGAN demonstrates powerful capacity for the inverse design of Voronoi lattices.

## 4. Conclusions

Herein, we developed a deep learning framework for the inverse design of voxelized Voronoi lattices. A 3D-CGAN neural network was trained using 10,000 randomly created Voronoi lattices and their labels (relative density and Young’s modulus), based on supervised learning. The trained 3D-CGAN was capable of rapidly generating 3D Voronoi lattices with the desired target relative density and Young’s modulus. Thus, this study demonstrates the potential application of Voronoi lattices in tissue engineering, where artificial scaffolds can be inversely generated using a given target relative density and Young’s modulus. We expect the scope of this study to be extended to the inverse design of architected cellular materials with other target properties by replacing the labels – for example, diffusivity, permeability, and conductivity – for the sake of energy storage and conservation [33,72,73]. Finally, although we only focused on a typical geometry (Voronoi lattices) in this study, the proposed approach has the potential to combine other geometries created using other methods, such as triply periodic minimal surfaces, spinodal architectures, and foams [30,31,64], to enable the inverse design of architected cellular materials inside and outside the material property space [57].

## Figures and Tables

**Figure A1.**
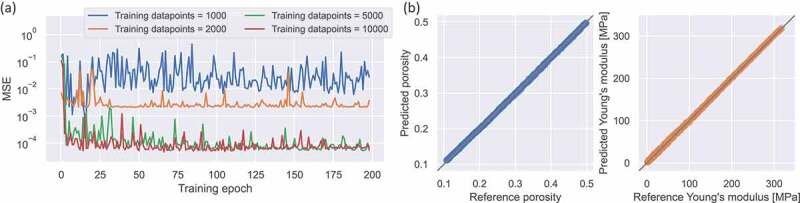
Performance of the calculator. (a) MSE versus training epoch using different numbers of datapoints. (b) Reference against calculator-predicted relative density and Young’s modulus.

## Appendix A. Details of 3D-CGAN

A 3D-CGAN is a framework used for the controllable generation of Voronoi lattices using adversarial process estimation. Three modules of deep neural networks (i.e., a generator, discriminator, and calculator) constitute the 3D-CGAN, and these modules are trained for different purpose. The generator generates 3D arrays of Voronoi lattices with target labels (relative density and Young’s modulus) from the latent space. It learns to deceive the discriminator and calculator simultaneously by generating geometries that look real and possess the desired target properties. The discriminator evaluates whether its input (a 3D array of a geometry) is generated by the generator or sampled from the “real” data. The calculator serves as an FEM simulator and predicts the relative density and Young’s modulus of a given input (3D array of geometries). The training process of the 3D-CGAN can be regarded as constituting three players in a minimax game, and the objective can be stated as follows:

(A1) $$\begin{array}{rl} & {\hat{\theta }}_{D}=\underset{\theta_{D}}{\arg\min}\{L_{D}(t_{D},D(X;\theta_{D}))\\ & \;\;\;\;\;\;\;\;\;\;\;\;+L_{D}(u_{D},D(G(Z,L;\theta_{G});\theta_{D}))\} \end{array}$$

(A2) $$\begin{array}{rl} & {\hat{\theta }}_{G}=\underset{\theta_{G}}{\arg\max}\{L_{D}(u_{D},D(G(Z,L;\theta_{G});\theta_{D}))\\ & \;\;\;\;\;\;\;\;\;\;\;\;-\alpha L_{C}(L,C(G(Z,L;\theta_{G});\theta_{C}))\} \end{array}$$

(A3) $$\begin{array}{c} {\hat{\theta }}_{C}=\underset{\theta_{C}}{\arg\max}\{L_{S}(L,S(X;\theta_{C}))\} \end{array}$$

where D, G, and C refer to the discriminator, generator, and calculator, respectively; L denotes the loss functions (binary cross-entropy function) of the three modules; and $t_{D}$ and $u_{D}$ denote the target labels. To mitigate the overconfidence of the 3D-CGAN, we penalized the discriminator and generator using label smoothing. This was accomplished by setting our target labels as random numbers: $t_{D}\in [0.7,1.2]$ and $u_{D}\in [0,0.3]$ [74, 75]. $X\in R^{n\times p}$ denotes the training set (3D arrays of voxelized Voronoi lattices) and was sampled randomly from the dataset with a batch size of 32. Here, θ denotes the sets of parameters of the three modules, $L\in R^{n\times l}$ denotes the labels of the input training set, X (i.e., normalized relative densities and Young’s moduli), $Z\in R^{n\times l}$ denotes the one-dimensional latent variable with a size of 128, and α is a learning parameter and was set to 0.1.

Adam optimization was implemented in the training process to optimize the parameters in the 3D-CGAN. The learning rates of the Adam optimizer were set to α = 0.0001 and $\beta_{1}=0.5$. The Wasserstein distance was used to improve the quality of the generated samples. The gradient penalty regularized the discriminator in the 3D-CGAN to assist the convergence, and its parameter was set to 100. The batch size for training was set to 32, and the epoch for training was set to 200. A dataset consisting of 10,000 datapoints was used to train the 3D-CGAN. The details of the three neural network structures are listed in Tables A1–A3. Thus, the generator consists of a fully connected layer, batch normalization, dropout, and a 3D transposed convolutional layer, and it uses activation functions including Leaky ReLU and tanh; the discriminator consists of a fully connected layer, dropout, and 3D convolutional layer and uses activation functions including Leaky ReLU and tanh; the calculator consists of six 3D convolution blocks with a 3D max pooling layer in each of them, and it finally consists of three fully connected layers. As the Voronoi lattices are triply periodic, we applied circular padding in the convolutional layers for both the up and down sampling processes.

The training process of the 3D-CGAN was conducted using TensorFlow on a single NVIDIA RTX A6000 graphic card on Linux system. The calculator was first trained using supervised learning, and its weights were used to train the discriminator and generator. The calculator was a linear regression model that used a 3D array of Voronoi lattices as an input and yielded the predicted relative densities and Young’s moduli as outputs. To investigate the effect of the number of datapoints on the performance of the calculator, we trained the calculator using different datasets. For each training dataset, 80% of the datapoints were used as the training set, and 20% of the datapoints were used as the testing set. It required approximately 10 h to train 200 epochs using a dataset consisting of 10,000 datapoints. Figure A1a compares the MSE of the testing set for different numbers of datapoints. It should be noted that it can be difficult for the calculator to converge if the datapoints are less than 1000. However, with an increase in the number of datapoints, the calculator converged, and the MSE decreased, indicating that an increasing number of datapoints was beneficial for the training. The MSE converged to near 0.0001, though it is hard to reduce it further, even bigger datapoints were utilized.

To save training time and resources, we selected 10,000 datapoints for the training process. A more quantitative evaluation of the calculator is shown in Figure A1b, wherein the reference and predicted values are compared in terms of the relative densities and Young’s moduli. The comparison between calculator-predicted and reference values showed an excellent agreement, proving the accuracy of the trained calculator. The well-trained calculator was saved as checkpoints and imported when training the generator and discriminator. Training the generator and discriminator was more time consuming, and it cost approximately 32 h to train 10,000 datapoints for 200 epochs. After the three modules were trained, the 3D-CGAN was used for the inverse design of voxelized Voronoi lattices.

**Table A1.** Neural network structure of the generator.

Neural network types	Kernel size	Resampling	Input array shape	Output array shape
Concatenate(Z, L)	-	-	128 + 2	130
Dense + BatchNormalization + Reshape	-	-	130	2 × 2 × 2 × 512
3D transposed convolution + BatchNormalization + Leaky ReLU + Dropout (0.3)	4 × 4	Up	2 × 2 × 2 × 512	4 × 4 × 4 × 256
3D transposed convolution + BatchNormalization + Leaky ReLU + Dropout (0.3)	4 × 4	Up	4 × 4 × 4 × 256	8 × 8 × 8 × 128
3D transposed convolution + BatchNormalization + Leaky ReLU + Dropout (0.3)	4 × 4	Up	8 × 8 × 8 × 128	16 × 16 × 16 × 64
3D transposed convolution + BatchNormalization + Leaky ReLU + Dropout (0.3)	4 × 4	Up	16 × 16 × 16 × 64	32 × 32 × 32 × 32
3D transposed convolution	4 × 4	Up	32 × 32 × 32 × 32	64 × 64 × 64 × 1
Sigmoid	-	-	64 × 64 × 64 × 1	64 × 64 × 64 × 1

**Table A2.** Neural network structure of the discriminator.

Neural network types	Kernel size	Resampling	Input array shape	Output array shape
Conv3D + LeakyReLU + Dropout (0.3)	4 × 4	Down	64 × 64 × 64 × 1	32 × 32 × 32 × 16
Conv3D + LeakyReLU + Dropout (0.3)	4 × 4	Down	32 × 32 × 32 × 16	16 × 16 × 16 × 32
Conv3D + LeakyReLU + Dropout (0.3)	4 × 4	Down	16 × 16 × 16 × 32	8 × 8 × 8 × 64
Conv3D + LeakyReLU + Dropout (0.3)	4 × 4	Down	8 × 8 × 8 × 64	4 × 4 × 4 × 128
Conv3D + LeakyReLU + Dropout (0.3)	4 × 4	Down	4 × 4 × 4 × 128	2 × 2 × 2 × 256
Conv3D + LeakyReLU + Dropout (0.3)	4 × 4	Down	2 × 2 × 2 × 256	1 × 1 × 1 × 512
Flatten	-	-	1 × 1 × 1 × 512	512
Dense	-	-	512	1

**Table A3.** Neural network structure of the calculator.

	Neural network types	Kernel/pool size	Resampling	Input array shape	Output array shape
Unit 1	Conv3D	3 × 3	-	64 × 64 × 64 × 1	64 × 64 × 64 × 16
	Conv3D	3 × 3	-	64 × 64 × 64 × 16	64 × 64 × 64 × 16
	3D max pooling	2 × 2	Down	64 × 64 × 64 × 16	32 × 32 × 32 × 16
Unit 2	Conv3D	3 × 3	-	32 × 32 × 32 × 16	32 × 32 × 32 × 32
	Conv3D	3 × 3	-	32 × 32 × 32 × 32	32 × 32 × 32 × 32
	3D max pooling	2 × 2	Down	32 × 32 × 32 × 32	16 × 16 × 16 × 32
Unit 3	Conv3D	3 × 3	-	16 × 16 × 16 × 32	16 × 16 × 16 × 64
	Conv3D	3 × 3	-	16 × 16 × 16 × 64	16 × 16 × 16 × 64
	3D max pooling	2 × 2	Down	16 × 16 × 16 × 64	8 × 8 × 8 × 64
Unit 4	Conv3D	3 × 3	-	8 × 8 × 8 × 64	8 × 8 × 8 × 128
	Conv3D	3 × 3	-	8 × 8 × 8 × 128	8 × 8 × 8 × 128
	3D max pooling	2 × 2	Down	8 × 8 × 8 × 128	4 × 4 × 4 × 128
Unit 5	Conv3D	3 × 3	-	4 × 4 × 4 × 128	4 × 4 × 4 × 256
	Conv3D	3 × 3	-	4 × 4 × 4 × 256	4 × 4 × 4 × 256
	3D max pooling	2 × 2	Down	4 × 4 × 4 × 256	2 × 2 × 2 × 256
Unit 6	Conv3D	3 × 3	-	2 × 2 × 2 × 256	2 × 2 × 2 × 512
	Conv3D	3 × 3	-	2 × 2 × 2 × 512	2 × 2 × 2 × 512
	3D max pooling	2 × 2	Down	2 × 2 × 2 × 512	1 × 1 × 1 × 512
	Flatten + Dense	-	-	1 × 1 × 1 × 512	256
	Dense			256	128
	Dense	-	-	128	2
